# Reduced Concentrations of NSE, S100*β*, A*β*, and Proinflammatory Cytokines in Elderly Patients Receiving Ultrasound-Guided Combined Lumbar Plexus-Sciatic Nerve Block during Hip Replacement

**DOI:** 10.1155/2022/1384609

**Published:** 2022-03-11

**Authors:** Yi Zhang, Liya Jiang, Yang Han

**Affiliations:** Chun'an Hospital of Traditional Chinese Medicine, No. 1 Xin'an West Road, Qiandaohu Town, Chun'an County, Zhejiang Province 311700, China

## Abstract

**Objective:**

The increase of hip fractures is related to the aging of the population, which has caused a huge medical burden in many countries. Hip replacement has been approved as a highly successful surgical intervention for the patients with hip fractures. Different anesthesia choices in the surgical intervention are associated with the prognosis of patients. This study focused on investigating the application of ultrasound-guided combined lumbar plexus-sciatic nerve block in elderly patients with hip fractures.

**Methods:**

In this retrospective study, 62 elderly patients received combined spinal-epidural anesthesia and 58 elderly patients underwent ultrasound-guided combined lumbar plexus-sciatic nerve block during the surgery. Hemodynamic monitoring including pulse oxygen saturation (SpO_2_), heart rate and blood pressure, the assessment of pain intensity using Visual Analogue Scale (VAS), cognitive function assessment through Montreal Cognitive Assessment (MoCA) and biomarkers consisting of serum levels of neuron specific-enolase (NSE), S100 beta protein (S100-*β*), and amyloid beta protein (A*β*), as well as immune function by interleukin-6 (IL-6), interleukin-1*β* (IL-1*β*), tumor necrosis factor-*α* (TNF-*α*), and high sensitivity C-reactive protein (hs-CRP) were detected in this study. Furthermore, length of hospital stay (LOS) and adverse reactions including hematoma, hypotension, nausea, and vomit were analyzed.

**Results:**

The findings indicated that comparing with the patients receiving combined spinal-epidural anesthesia, those undergoing ultrasound-guided combined lumbar plexus-sciatic nerve block showed significantly lower level of heart rate, higher level of SpO_2_, and lower level of diastolic pressure and systolic pressure at 5 minutes and 30 minutes after anesthesia and after surgery (*P* < 0.05), indicated obviously lower VAS score at 12, 24, and 48 hours after surgery (*P* < 0.05), and revealed higher MoCA score at 12 days after surgery (*P* < 0.05). A significantly higher level of NSE, S100*β*, A*β*, IL-6, IL-1*β*, TNF-*α,* and hs-CRP was revealed in the two groups receiving different anesthesia methods at 10 days after surgery compared with that before surgery (*P* < 0.05). However, the patients receiving ultrasound-guided combined lumbar plexus-sciatic nerve block had obviously lower expression of NSE, S100*β*, A*β*, IL-6, IL-1*β*, TNF-*α,* and hs-CRP compared with the group accepting combined spinal-epidural anesthesia (*P* < 0.05). The two groups indicated no significant difference in incidence of hypotension and vomit, etc. (*P* < 0.05), but showed remarkable difference referring to total incidence of adverse reactions and LOS (*P* < 0.05).

**Conclusion:**

The application of ultrasound-guided combined lumbar plexus-sciatic nerve block in hip replacement contributes to the stability of hemodynamics and alleviation of postoperative pain intensity. It can reduce cognitive and immune impairment of the elderly patients with hip fractures.

## 1. Introduction 

Elderly people are prone to experience hip fractures, which has caused high incidence of mortality due to decline in overall health and poor prognosis. It has been reported that the elderly patients with hip fractures were older than 65 years and the average age was 80 years, of which 80% are female patients [1, 2]. Women over the age of 85 are 10 times more likely to have hip fractures than women aged 60 to 69 [3]. Hip replacement is a standard surgical intervention for the patients with hip fractures. However, some reported mentioned that this intervention might lead to specific complications, especially dislocation [4]. Fortunately, anterolateral approach has been confirmed as an effective way to reduce the risk of hip dislocation in the population with hip fracture [5, 6].

The patients undergoing hip replacement will bear a considerable and persistent burden caused by the disease, affecting their quality of life, and accumulated evidence indicated bad quality of life is related to cognitive impairment [7, 8]. Postoperative cognitive dysfunction (POCD) refers to the decline of cognitive function objectively measured after anesthesia and surgery. POCD in the elderly after anesthesia and surgery has been well described [9]. POCD is generally correlated with cerebral injury. The studies on animals and humans demonstrated neuron specific-enolase (NSE), S100 beta protein (S100-*β*), and amyloid beta protein (A*β*) have been approved as potential biochemical markers of cerebral injury [10, 11]. Cerebral injury leads to an increase in the serum concentration of these biochemical markers [12]. Different anesthesia methods have different effects on the immune function of perioperative patients and even related to the prognosis of patients. Proinflammatory markers, such as interleukin-6 (IL-6), interleukin-1*β* (IL-1*β*), tumor necrosis factor-*α* (TNF-*α*), and C-reactive protein (CRP), are highly associated with immune function. Elevated concentration of proinflammatory markers will reduce immune function [13, 14]. Therefore, the choice of anesthesia for elderly patients with hip fractures undergoing hip replacement plays an essential role in their inflammatory reaction and cognitive function.

The role of ultrasound-guided lumbar plexus block and ultrasound-guided sciatic nerve block alone has been well established in hip surgery [15, 16]. However, few studies were founded on the combination of ultrasound-guided lumbar plexus block and sciatic nerve block in the hip replacement. This study retrospectively analyzed the effects of ultrasound-guided combined lumbar plexus-sciatic nerve block on cognitive function and inflammatory reaction in the elderly patients with hip fractures undergoing hip replacement.

## 2. Materials and Methods

### 2.1. Subject Assignments

This study retrospectively analyzed the conditions of 120 elderly patients with hip fractures who underwent hip replacement in our hospital from January 2019 to January 2021. In this study, 62 cases received combined spinal-epidural anesthesia and the remaining 58 patients were treated with ultrasound-guided combined lumbar plexus-sciatic nerve block during the surgery. The patients undergoing combined spinal-epidural anesthesia included 30 females and 32 males, who ranged from 61 to 75 years old with an average age of (68 ± 1.68) years and contained 29 cases of the American Society of Anaesthesiologists (ASA) [17] grade II and 33 cases of ASA grade III. There were 28 females and 30 males (ASA grade II 29 cases, ASA grade III 29 cases) receiving ultrasound-guided combined lumbar plexus-sciatic nerve block in the surgery, and they aged from 63 to 74 years old with an average age of (66 ± 1.36) years. No significant difference was found in the terms of gender, age, and ASA grade, etc., among the patients with different anesthesia methods (*P* > 0.05).

All the patients involved in the study were diagnosed with hip fractures and classified into ASA grade II or grade III and underwent hip replacement. Furthermore, the patients' age was no less than 60 years old. Those diagnosed patients must be excluded in the study if they were allergic to anesthetic drugs, had infection in the area to be anesthetized, had severe organic lesions of the heart, kidney, and liver, accompanied by nervous system diseases or severe cognitive impairment, and suffered from dysfunction of blood coagulation and serious respiratory diseases. This study was accepted by all the studied patients and approved by our Hospital Ethics Committee.

### 2.2. Treatment Protocol

Establishment of vascular access was applied to each patient and vital signs including blood pressure, electrocardiogram, and blood oxygen saturation, etc., were monitored. Furthermore, all the fasting patients were given intravenous drip of sodium lactate ringer's injection (6–8 ml/kg), fentanyl citrate (1 uG/kg), and midazolam (0.1 mg/kg) before surgery. Combined spinal-epidural anesthesia was carried out through intraspinal anesthesia. In short, the patient maintained lateral position and received routine disinfection firstly, followed by the puncture of subarachnoid space in L3-L4 intervertebral space, then 2 ml of 0.75% bupivacaine hydrochloride injection (specification: 5 ml: 37.5 mg, National Medicine Permission No. H20056442, Shanghai Zhaohui Pharmaceutical Co., Ltd., China) and 1 ml of 10% glucose were injected to the subarachnoid space. The epidural catheter was placed for each patient finally. 2 to 5 ml of 0.75% ropivacaine was injected through the epidural catheter according to the patient's situation during the surgery. Lumbar plexus and sciatic nerve block were performed under ultrasound guidance. The ultrasound systems (ACUSON P300, Siemens, Germany) were used to scan the horizontal plane of L4 with the parameters of 2.0 to 5.0 MHz while the patient maintained lateral position. After the puncture point was determined, the local infiltration anesthesia was carried out with 1% lidocaine hydrochloride injection, followed by the application of epidural puncture in the middle of the spine near the L4 point guided by ultrasound. The puncture was completed until the needle tip entered the L4 nerve root, and 20 ml of 0.5% ropivacaine hydrochloride was injected into the puncture point in the absence of cerebrospinal fluid. Connecting line between the ischial tuberosity and the greater trochanter was located by ultrasound scanning, and high-echo characterized by triangle or ellipse was found in the sciatic nerve. Finally, 20 ml of 0.5% ropivacaine injection was applied to the sciatic nerve punctured.

### 2.3. Outcome Measures

Pulse oxygen saturation (SpO_2_), heart rate, and blood pressure were monitored in perioperative period. The data of these outcome measures before anesthesia, 5 minutes and 30 minutes after anesthesia, and after surgery were analyzed to evaluate hemodynamics.

Visual Analogue Scale (VAS) is a scale which is developed to obtain more variable measurements, which measures potential features using a line continuum instead of the five or seven categories used by Likert-type scale [18]. VAS is widely used to measure pain intensity after surgery. The pain intensity of each patient in this study was assessed by VAS at 12, 24, and 48 hours after surgery. The pain intensity was positively correlated with the score. Montreal Cognitive Assessment (MoCA) was conducted before and 12 days after surgery. MoCA included 11 assessment items in the 8 cognitive fields, consisting of concentration, executive function, memory, language, visual structure skills, abstract thinking, calculation, and orientation. The total score is 30 points and the score no less than 26 points was considered as normal cognition.

5 ml of fasting venous blood was taken from each patient before surgery and 10 days after surgery and placed steadily for 2 hours before centrifugation. The centrifugation was performed at the speed of 3000 rpm for 15 minutes to obtain serum. The serum levels of NSE, S100-*β,* and A*β*, as well as IL-6, IL-1*β*, TNF-*α*, and high sensitivity C-reactive protein (hs-CRP) were detected by enzyme-linked immunosorbent assay (ELISA) reader (RT-6100, Rayto Life and Analytical Sciences Co., Ltd., China) before and 10 days after surgery.

Adverse reactions including hematoma, hypotension, nausea and vomit, and length of hospital stay (LOS) which were recorded and analyzed.

### 2.4. Data Processing

SPSS 22.0 statistical software was used to analyze and process the data, the count data were expressed as percentage and analyzed by chi-square test, and the measurement data were described as mean ± standard deviation using *T* test. *P* < 0.05 indicated the difference was statistically significant.

## 3. Results

Ultrasound-guided combined lumbar plexus-sciatic nerve block contributed to the stability of hemodynamics.

No significant difference was revealed in the SpO_2,_ heart rate, and blood pressure between different treatment groups before anesthesia (*P* > 0.05). However, comparing with the patients receiving combined spinal-epidural anesthesia, the patients undergoing ultrasound-guided combined lumbar plexus-sciatic nerve block showed remarkably lower level of heart rate, higher level of SpO_2_, and lower level of diastolic pressure and systolic pressure at 5 minutes and 30 minutes after anesthesia and after surgery (*P* < 0.05). The details are listed in Table 1.

### 3.1. Ultrasound-Guided Combined Lumbar Plexus-Sciatic Nerve Block Relieved Postoperative Pain Intensity

As listed in Table 2, the VAS scores of the two groups receiving different anesthesia methods decreased significantly at 12, 24, and 48 hours after surgery compared with that before surgery (*P* < 0.05). The VAS score of the two groups remarkably decreased with time, and the VAS score at 12, 24, and 48 hours after surgery was obviously lower in the patients accepted ultrasound-guided combined lumbar plexus-sciatic nerve block compared with those undergoing spinal-epidural anesthesia (*P* < 0.05).

### 3.2. Ultrasound-Guided Combined Lumbar Plexus-Sciatic Nerve Block Alleviated Cognitive Impairment

In order to evaluate the cognitive impairment caused by different anesthesia methods, MoCA was applied to each patient before and 12 days after surgery. It was found that there was no significant difference in MoCA score between the two groups before surgery (*P* > 0.05). Compared with the score before surgery, obviously lower MoCA score revealed in both groups at 12 days after surgery (*P* < 0.05), but the patients undergoing ultrasound-guided combined lumbar plexus-sciatic nerve block showed higher MoCA score than those receiving combined spinal-epidural anesthesia (*P* < 0.05, Table 3).

Next, serum levels of NSE, S100*β*, and A*β* were compared between the two groups before and 10 days after surgery to access cognitive function. The results demonstrated slight difference in these biomarkers was showed between the two groups before surgery (*P* > 0.05). However, significantly higher levels of NSE, S100*β*, and A*β* were revealed in the two groups receiving different anesthesia methods at 10 days after surgery compared with that before surgery (*P* < 0.05). Furthermore, at 10 days after surgery, the group receiving ultrasound-guided combined lumbar plexus-sciatic nerve block had lower expression of NSE, S100*β*, and A*β* compared with the group accepting combined spinal-epidural anesthesia (*P* < 0.05, Table 4).

### 3.3. Ultrasound-Guided Combined Lumbar Plexus-Sciatic Nerve Block Prevented Inflammatory Response

ELISA results showed that the expression of IL-6, IL-1*β*, TNF-*α*, and hs-CRP increased significantly at 10 days after surgery in the two groups compared with these before surgery (*P* < 0.05). At 10 days after surgery, significant lower expression of IL-6, IL-1*β*, TNF-*α*, and hs-CRP was revealed in the patients accepting ultrasound-guided combined lumbar plexus-sciatic nerve block compared with the patients receiving combined spinal-epidural anesthesia (*P* < 0.05, Table 5).

### 3.4. Ultrasound-Guided Combined Lumbar Plexus-Sciatic Nerve Block Reduced Adverse Reactions and LOS

Anesthetic drugs and methods during surgery are associated with adverse reactions. Adverse reactions such as hematoma [19], hypotension [20], and nausea and vomit [21] commonly occurred after anesthesia. This study analyzed the incidence of hematoma, hypotension, nausea, and vomit after surgery. It was found that there was no significant difference in single adverse reaction between the two groups (*P* > 0.05). However, the total incidence of adverse reactions was significantly different between the two groups (*P* < 0.05, Table 6). Additionally, the group undergoing ultrasound-guided combined lumbar plexus-sciatic nerve block showed shorter LOS (*P* < 0.05).

## 4. Discussion

Hip fractures pose a significant burden on patients' health and the medical system. It is estimated that the resulting socio-economic costs account for 0.1% of the global disease burden [22] and nearly 30% of patients died within the first year after illness [23]. Hip fractures are commonly seen in elderly population. The prevalence of hip fractures is highly associated with age and gender. 20% of women and 10% of men have a lifetime prevalence of hip fractures [3]. Choice of treatment is decided by the general physical and psychological abilities of these patients.

In this study, the elderly patients with hip fractures underwent hip replacement but with different types of anesthesia. Some of the patients received combined spinal-epidural anesthesia and the others accepted ultrasound-guided combined lumbar plexus-sciatic nerve block. The study analyzed the effects of these two kinds of anesthesia on hemodynamic stability, cognitive and immune function, and adverse reactions in the elderly patients. Previous studies indicated peripheral regional anesthesia for hip surgery is correlated with better haemodynamic stability and reduce of complications compared with general anesthesia or spinal anesthesia [24, 25]. The present study measured SpO_2_, heart rate, and blood pressure for all patients and found that at 5 minutes and 30 minutes after anesthesia and after surgery, remarkably lower level of heart rate, higher level of SpO_2_, and lower level of diastolic pressure and systolic pressure were revealed in the patients undergoing ultrasound-guided combined lumbar plexus-sciatic nerve block compared with the patients receiving combined spinal-epidural anesthesia. Lumbar plexus block can be performed with an ultrasound probe applied to a paravertebral location. The needle can be displayed in real time in the peripheral nerve structure through sciatic nerve block guided by ultrasound, which improves the accuracy and safety of surgery. VAS is a popular tool for the measurement of pain and commonly recommended for assessment of pain intensity in the area of cancer [26]. At 12, 24, and 48 hours after surgery, the patients who accepted ultrasound-guided combined lumbar plexus-sciatic nerve block showed obviously lower VAS score compared with those treated with other anesthetic techniques. It suggested that the pain relief may be related to the accuracy of puncture position under ultrasound guidance. It was reported up to 65% of patients undergoing surgery sustained cognitive dysfunction at hospital discharge. Neuro-inflammation caused by surgery or anesthesia is closely related to the development of POCD [27]. It is generally believed that cognitive function will decline with age, and any changes following anesthesia and surgery are associated with preoperative cognitive function. Therefore, this study compared the serum levels of NSE, S100*β*, and A*β* in the two groups before surgery. The results demonstrated there was no significant difference in levels of NSE, S100*β*, and A*β* between the two groups before surgery. NSE, S100*β*, and A*β* are useful biomarkers for assessing neuronal injury. Expressions of NSE, S100*β*, and A*β* elevate following surgery in humans and animals [10, 28]. At 10 days after surgery, these levels increased remarkably in both groups, but the group receiving ultrasound-guided combined lumbar plexus-sciatic nerve block had obviously lower expression of NSE, S100*β*, and A*β* compared with the other group. Additionally, the patients undergoing ultrasound-guided combined lumbar plexus-sciatic nerve block showed higher MoCA score than those receiving combined spinal-epidural anesthesia. MoCA is an effective screening tool to distinguish mild cognitive impairment patients from normal people. It shows high sensitivity and specificity in Alzheimer's disease, frontotemporal dementia, and Parkinson's disease-related dementia [29]. The outcomes revealed ultrasound-guided combined lumbar plexus-sciatic nerve block alleviated cognitive impairment in elderly patients.

Surgery and anesthesia can cause strong systemic inflammatory response and activation of immune system. Inflammatory response system is driven by proinflammatory cytokines produced by macrophages, T cells, and natural killer cells in response to immune activation [30]. Enormous studies have manifested increased levels of proinflammatory cytokines such as CRP, IL-1*β*, IL-6, and TNF-*α* in elderly patients with depression ([31] and the patients underwent surgery) [32, 33]. ELISA results in this retrospective study indicated serum levels of IL-6, IL-1*β*, TNF-*α*, and hs-CRP increased significantly at 10 days after surgery in the two groups. However, the patients accepted ultrasound-guided combined lumbar plexus-sciatic nerve block showed significant lower expression of these proinflammatory cytokines, suggesting ultrasound-guided combined lumbar plexus-sciatic nerve block contributed to the reduce of immune function damage. Furthermore, this study revealed significant difference in total incidence of adverse reactions between the two groups. Comparing with those receiving combined spinal-epidural anesthesia, the group undergoing ultrasound-guided combined lumbar plexus-sciatic nerve block had much lower total incidence of adverse reactions.

This study analyzed the role of ultrasound-guided combined lumbar plexus-sciatic nerve block in elderly patients undergoing surgery and found that ultrasound-guided combined lumbar plexus-sciatic nerve block contributed to alleviate the damage of cognitive and relieve inflammatory response. However, this study with small sample size may reduce the reliability of data.

## Figures and Tables

**Table 1:** SpO_2,_ heart rate, and blood pressure in the two groups.

Treatments	*N*		Before anesthesia	5 minutes after anesthesia	30 minutes after anesthesia	After surgery
Ultrasound-guided combined lumbar plexus-sciatic nerve block	58	Heart rate (time/min)	81.52 ± 4.91	92.60 ± 5.20^*∗*^	85.04 ± 7.07^*∗*^	80.00 ± 6.99^*∗*^
		SPO_2_/%	96.17 ± 1.42	98.72 ± 1.38^*∗*^	97.10 ± 1.98^*∗*^	97.23 ± 0.98^*∗*^
		Diastolic pressure (mmHg)	99.29 ± 5.32	100.16 ± 5.38^*∗*^	96.84 ± 3.45^*∗*^	94.85 ± 6.88^*∗*^
		Systolic pressure (mmHg)	143.37 ± 6.10	149.45 ± 5^*∗*^	140.43 ± 6.18^*∗*^	131.54 ± 5.48^*∗*^

Combined spinal-epidural anesthesia	62	Heart rate (time/min)	81.16 ± 6.42	99.69 ± 5.89	94.09 ± 6.77	87.93 ± 6.11
		SPO_2_/%	96.06 ± 1.19	94.14 ± 0.96	92.82 ± 1.93	95.50 ± 0.93
		Diastolic pressure (mmHg)	97.03 ± 5.44	108.71 ± 3.60	101.70 ± 3.23	100.72 ± 3.56
		Systolic pressure (mmHg)	142.90 ± 6.90	155.34 ± 6.09	148.18 ± 5.61	137.76 ± 6.99

Comparing with combined spinal-epidural anesthesia, ^*∗*^ indicates *P* < 0.05.

**Table 2:** VAS score in the two groups.

Treatments	*N*	3 h after surgery	12 h after surgery	24 h after surgery	*F*	*P*
Ultrasound-guided combined lumbar plexus-sciatic nerve block	58	3.00 ± 1.31	2.63 ± 0.99	1.69 ± 0.74	24.67	<0.0001
Combined spinal-epidural anesthesia	62	3.79 ± 1.59	3.07 ± 1.05	2.13 ± 0.66	31.81	<0.0001
*T*		2.959	2.358	3.442		
*P*		0.004	0.020	<0.001		

**Table 3:** MoCA score in the two groups.

Treatments	*N*	Before surgery	12 days after surgery
Ultrasound-guided combined lumbar plexus-sciatic nerve block	58	27.75 ± 0.96	25.01 ± 1.32^*a∗*^
Combined spinal-epidural anesthesia	62	27.82 ± 0.56	23.88 ± 0.78^a^
*T*		0.492	5.753
*P*		0.624	<0.0001

Comparing with combined spinal-epidural anesthesia, ^*∗*^indicates *P* < 0.05. Comparing with that before surgery, ^a^indicates *P* < 0.05.

**Table 4:** Expression of biomarkers of cognitive function in the two groups.

Treatments	*N*	NSE		S100*β*		A*β*	
		Before surgery	10 days after surgery	Before surgery	10 days after surgery	Before surgery	10 days after surgery
Ultrasound-guided combined lumbar plexus-sciatic nerve block	58	8.78 ± 1.55	10.23 ± 0.26^*a∗*^	237.24 ± 33.31	267.37 ± 41.28^*a∗*^	259.15 ± 30.50	286.81 ± 21.75^*a∗*^
Combined spinal-epidural anesthesia	62	9.09 ± 1.93	10.60 ± 0.22^a^	242.63 ± 26.83	283.93 ± 26.05^a^	256.27 ± 30.25	298.28 ± 37.60^a^
*T*		0.966	8.434	0.979	2.646	0.519	2.027
*P*		0.336	<0.0001	0.330	0.009	0.605	0.045

^*∗*^ indicates *P* < 0.05 compared with combined spinal-epidural anesthesia; ^a^ indicates *P* < 0.05 compared with that before surgery.

**Table 5:** Proinflammatory cytokine levels in the two groups.

Treatments	*N*	IL-6		TNF-*α*		IL-1*β*		Hs-CRP	
		Before surgery	10 days after surgery	Before surgery	10 days after surgery	Before surgery	10 days after surgery	Before surgery	10 days after surgery
Ultrasound-guided combined lumbar plexus-sciatic nerve block	58	52.11 ± 12.01	102.30 ± 36.98^*a∗*^	48.35 ± 13.47	80.88 ± 22.78^*a∗*^	35.72 ± 3.82	45.54 ± 5.68^*a∗*^	18.42 ± 0.19	23.93 ± 2.60^*a∗*^

Combined spinal-epidural anesthesia	62	53.86 ± 11.85	124.53 ± 41.31^a^	44.17 ± 11.11	101.56 ± 27.04^a^	35.66 ± 4.83	50.90 ± 5.59^a^	18.5 ± 0.29	28.02 ± 2.17^a^

*T*		0.803	3.098	1.859	4.515	0.075	5.208	1.774	9.378

*P*		0.4235	0.0024	0.0655	<0.0001	0.940	<0.0001	0.079	<0.0001

^*∗*^indicates *P* < 0.05 compared with combined spinal-epidural anesthesia; ^a^indicates *P* < 0.05 compared with that before surgery.

**Table 6:** Adverse reactions and LOS in the two groups after surgery.

Treatments	Hypotension (*n)*	Hematoma/*n*	Nausea/*n*	Vomit/*n*	Total rate (*n*, %)	LOS
Ultrasound-guided combined lumbar plexus-sciatic nerve block	1	0	0	0	1 (1.72%)	11.80 ± 1.17
Combined spinal-epidural anesthesia	3	2	1	1	7 (11.29%)	14.17 ± 1.25
*Z*	0.9498	1.379	0.9713	0.9713	2.099	10.70
*P*	0.3422	0.1678	0.3314	0.3314	0.0358	<0.0001

## Data Availability

The data used to support the findings of this study are included within the article.
